# Parkin is activated by PINK1-dependent phosphorylation of ubiquitin at Ser^65^

**DOI:** 10.1042/BJ20140334

**Published:** 2014-04-25

**Authors:** Agne Kazlauskaite, Chandana Kondapalli, Robert Gourlay, David G. Campbell, Maria Stella Ritorto, Kay Hofmann, Dario R. Alessi, Axel Knebel, Matthias Trost, Miratul M. K. Muqit

**Affiliations:** *MRC Protein Phosphorylation and Ubiquitylation Unit, University of Dundee, Dundee, Scotland, U.K.; †Institute for Genetics, University of Cologne, Cologne, Germany; ‡College of Medicine, Dentistry & Nursing, University of Dundee, Dundee, Scotland, U.K.

**Keywords:** Parkin, Parkinson’s disease, phosphorylation, PTEN (phosphatase and tensin homologue deleted on chromosome 10)-induced putative kinase 1 (PINK1), ubiquitin, CCCP, carbonyl cyanide *m*-chlorophenylhydrazone, CDK2, cyclin-dependent kinase 2, GSK3β, glycogen synthase kinase-3β, HEK, human embryonic kidney, HOIL1, haem-oxidized IRP2 (iron-regulatory protein 2) ubiquitin ligase 1, HRP, horseradish peroxidase, IKK, IκB (inhibitor of nuclear factor κB) kinase, ISG15, interferon-induced 17 kDa protein, MBP, maltose-binding protein, MLK1, mixed lineage kinase 1, Nedd8, neural-precursor-cell-expressed developmentally down-regulated 8, Ni-NTA, Ni^2+^-nitrilotriacetate, NUAK1, NUAK family SNF1-like kinase 1, OTU1, OTU (ovarian tumour) domain-containing protein 1, PD, Parkinson’s disease, PINK1, PTEN (phosphatase and tensin homologue deleted on chromosome 10)-induced putative kinase 1, PLK1, Polo-like kinase 1, SILAC, stable isotope labelling by amino acids in cell culture, SUMO, small ubiquitin-related modifier, TCEP, tris-(2-carboxyethyl)phosphine, TcPINK1, *Tribolium castaneum* PINK1, Ubl, ubiquitin-like

## Abstract

We have previously reported that the Parkinson's disease-associated kinase PINK1 (PTEN-induced putative kinase 1) is activated by mitochondrial depolarization and stimulates the Parkin E3 ligase by phosphorylating Ser^65^ within its Ubl (ubiquitin-like) domain. Using phosphoproteomic analysis, we identified a novel ubiquitin phosphopeptide phosphorylated at Ser^65^ that was enriched 14-fold in HEK (human embryonic kidney)-293 cells overexpressing wild-type PINK1 stimulated with the mitochondrial uncoupling agent CCCP (carbonyl cyanide *m*-chlorophenylhydrazone), to activate PINK1, compared with cells expressing kinase-inactive PINK1. Ser^65^ in ubiquitin lies in a similar motif to Ser^65^ in the Ubl domain of Parkin. Remarkably, PINK1 directly phosphorylates Ser^65^ of ubiquitin *in vitro*. We undertook a series of experiments that provide striking evidence that Ser^65^-phosphorylated ubiquitin (ubiquitin^Phospho−Ser65^) functions as a critical activator of Parkin. First, we demonstrate that a fragment of Parkin lacking the Ubl domain encompassing Ser^65^ (ΔUbl-Parkin) is robustly activated by ubiquitin^Phospho−Ser65^, but not by non-phosphorylated ubiquitin. Secondly, we find that the isolated Parkin Ubl domain phosphorylated at Ser^65^ (Ubl^Phospho−Ser65^) can also activate ΔUbl-Parkin similarly to ubiquitin^Phospho−Ser65^. Thirdly, we establish that ubiquitin^Phospho−Ser65^, but not non-phosphorylated ubiquitin or Ubl^Phospho−Ser65^, activates full-length wild-type Parkin as well as the non-phosphorylatable S65A Parkin mutant. Fourthly, we provide evidence that optimal activation of full-length Parkin E3 ligase is dependent on PINK1-mediated phosphorylation of both Parkin at Ser^65^ and ubiquitin at Ser^65^, since only mutation of both proteins at Ser^65^ completely abolishes Parkin activation. In conclusion, the findings of the present study reveal that PINK1 controls Parkin E3 ligase activity not only by phosphorylating Parkin at Ser^65^, but also by phosphorylating ubiquitin at Ser^65^. We propose that phosphorylation of Parkin at Ser^65^ serves to prime the E3 ligase enzyme for activation by ubiquitin^Phospho−Ser65^, suggesting that small molecules that mimic ubiquitin^Phospho−Ser65^ could hold promise as novel therapies for Parkinson's disease.

## Abstract 2

We describe a novel and unexpected mechanism by which PINK1 protein kinase activates Parkin E3 ligase. We show that PINK1 phosphorylates ubiquitin at Ser^65^ and that phosphorylated ubiquitin acts as a direct activator of Parkin.

## INTRODUCTION

Mutations in PINK1 [PTEN (phosphatase and tensin homologue deleted on chromosome 10)-induced putative kinase 1] and Parkin are associated with early-onset autosomal-recessive PD (Parkinson's disease) [1,2]. Several lines of evidence indicate that these enzymes function in a common signalling pathway. For example, patients bearing PINK1 or Parkin mutations share a similar phenotype [3–5], and compelling genetic studies in *Drosophila melanogaster* suggests that PINK1 acts upstream of Parkin [6–8]. Furthermore, PINK1 has been reported to be required for Parkin recruitment to mitochondria upon mitochondrial membrane depolarization in mammalian cell lines [9–12]. We recently found that PINK1 can phosphorylate Parkin directly at a highly conserved residue, Ser^65^, that lies within the Ubl (ubiquitin-like) domain of Parkin and demonstrated that phosphorylation stimulates Parkin E3 ligase activity [13]. Recent high-resolution crystal structures of Parkin lacking the Ubl domain suggest that Parkin is autoinhibited; however, the studies do not shed any light on the mechanism of how Ser^65^ phosphorylation triggers conformational change and activation of Parkin [14–16].

PINK1 is unique among all protein kinases since it possesses a N-terminal targeting motif that localizes it to the mitochondria where it undergoes sequential cleavage by mitochondrial processing protease and the rhomboid protease PARL (presenilin-associated rhomboid-like protein, mitochondrial) followed by rapid degradation by the N-end rule pathway [17]. In response to mitochondrial membrane depolarization, PINK1 becomes stabilized at the mitochondria where it becomes activated and autophosphorylates at Thr^257^ and phosphorylates Parkin at Ser^65^ [13]. Protein kinases are able to phosphorylate anywhere from one to a large number of substrates [18]; however, it is unknown whether PINK1 can phosphorylate additional substrates in the mitochondria upon mitochondrial depolarization.

In the present study we have identified a novel ubiquitin phosphopeptide phosphorylated at Ser^65^ that was enriched significantly in HEK (human embryonic kidney)-293 cells expressing wild-type PINK1 upon PINK1 activation by the mitochondrial uncoupler CCCP (carbonyl cyanide *m*-chlorophenylhydrazone), compared with cells expressing kinase-inactive PINK1. We demonstrate that PINK1 can directly phosphorylate ubiquitin specifically at Ser^65^. Using a Ubl-deleted fragment of Parkin, ΔUbl-Parkin (which lacks the Parkin Ser^65^ site), we observe robust enhancement in Parkin E3 ligase activity upon phosphorylation by wild-type, but not kinase-inactive, PINK1 and this effect on activity is abolished if we use an S65A ubiquitin mutant in our assays. To obtain definitive insights into the role of PINK1-induced phosphorylation of ubiquitin in regulating Parkin activity, we have purified Ser^65^-phosphorylated ubiquitin (ubiquitin^Phospho−Ser65^) to homogeneity and demonstrate that in stark contrast with non-phosphorylated ubiquitin, it is capable of activating both full-length wild-type and ΔUbl-Parkin E3 ligase activity. The present study provides fundamental novel mechanistic insights into how PINK1 activates Parkin E3 ligase activity and suggests a dual regulatory mechanism of Parkin E3 ligase activity in which PINK1-dependent phosphorylation of Parkin at Ser^65^ is required to prime Parkin for activation by ubiquitin^Phospho−Ser65^.

## MATERIALS AND METHODS

### Materials

[γ-^32^P]ATP was from PerkinElmer. All mutagenesis was carried out using the QuikChange® site-directed mutagenesis method (Stratagene) with KOD polymerase (Novagen). All DNA constructs were verified by DNA sequencing, which was performed by the Sequencing Service, College of Life Sciences, University of Dundee, Dundee, Scotland, U.K., using DYEnamic ET terminator chemistry (GE Healthcare) on Applied Biosystems automated DNA sequencers. DNA for bacterial protein expression was transformed into *Escherichia coli* BL21-CodonPlus (DE3)-RIL cells (Stratagene). All cDNA plasmids, antibodies and recombinant proteins generated for the present study are available on request through our reagents website (http:s://mrcppureagents.dundee.ac.uk/).

### Antibodies

An antigen affinity-purified sheep anti-SUMO-1 (small ubiquitin-related modifier 1) antibody was a gift from Professor Ron Hay (College of Life Sciences, University of Dundee, Dundee, Scotland, U.K.). An anti-Parkin mouse monoclonal antibody was obtained from Santa Cruz Biotechnology. An HRP (horseradish peroxidase)-conjugated anti-FLAG antibody was obtained from Sigma.

### Immunoblotting

Samples were subjected to SDS/PAGE (4–12% gels) and were transferred on to nitrocellulose membranes. Membranes were blocked for 1 h in TBST [Tris-buffered saline (50 mM Tris/HCl and 150 mM NaCl, pH 7.5) with 0.1% Tween 20] containing 5% (w/v) non-fat dried skimmed milk powder. Membranes were probed with the indicated antibodies in TBST containing 5% (w/v) non-fat dried skimmed milk powder overnight at 4°C. Detection was performed using HRP-conjugated secondary antibodies and enhanced chemiluminescence reagent.

### Cell culture

Flp-In T-Rex stable cell lines were cultured using DMEM (Dulbecco's modified Eagle's medium) supplemented with 10% FBS, 2 mM L-glutamine, 1×penicillin/streptomycin, 15 μg/ml blasticidin and 100 μg/ml hygromycin. Cultures were induced to express protein by the addition of 0.1 μg/ml doxycycline in the medium for 24 h. To uncouple mitochondria, cells were treated with 10 μM CCCP (Sigma) dissolved in DMSO for 3 h.

### Identification of Ser^65^ phosphorylation of ubiquitin by MS

We undertook a SILAC (stable isotope labelling by amino acids in cell culture)-based quantitative phosphoproteomic screen in Flp-In T-Rex HEK-293 cells stably expressing FLAG-empty (L), wild-type (H) or kinase-inactive (M) PINK1–FLAG. Cells were stimulated with 10 μM CCCP for 3 h and homogenized in 8.55% (w/v) sucrose and 3 mM imidazole (pH 7.4) (supplemented with protease and phosphatase inhibitor cocktail from Roche, and benzonase from Roche). Mitochondria-containing membrane fractions were enriched by ultracentrifugation and solubilized in 1% RapiGest™ (Waters). Lysates were mixed from each cell condition at 1:1:1 before being subjected to tryptic digestion. Digested peptides were subjected to TiO_2_ phosphopeptide enrichment [19,20] and analysed by LC–MS on an Orbitrap Velos Pro (Thermo Fisher). Data were analysed using Maxquant 1.3.0.5 [21] and Xcalibur software (Thermo Fisher). More details and the full analysis of the screen are available on request (C. Kondapalli, B. Dill, J. Proctor, A. Kazlauskaite, M. Trost and M. Muqit, unpublished work).

### *In vitro* ubiquitylation assays

Wild-type or ΔUbl-Parkin (residues 80–465) (2 μg) was initially incubated with 1 μg of *E. coli*-expressed wild-type or kinase-inactive (D359A) MBP (maltose-binding protein)–TcPINK1 (*Tribolium castaneum* PINK1) in a reaction volume of 25 μl [50 mM Tris/HCl (pH 7.5), 0.1 mM EGTA, 10 mM magnesium acetate, 1% 2-mercaptoethanol and 0.1 mM ATP]. Kinase assays were carried out at 30°C for 60 min followed by addition of ubiquitylation assay components and Mastermix to a final volume of 50 μl [50 mM Tris/HCl (pH 7.5), 0.05 mM EGTA, 10 mM MgCl_2_, 0.5% 2-mercaptoethanol, 0.12 μM human recombinant E1 purified from the Sf21 insect cell line, 1 μM human recombinant UbcH7 and 2 μg of His_6_–SUMO-Miro1 both purified from *E. coli*, 0.05 mM FLAG–ubiquitin (Boston Biochem) and 2 mM ATP]. Ubiquitylation reactions were carried out at 30°C for 60 min and terminated by the addition of SDS sample buffer. For all assays, reaction mixtures were resolved by SDS/PAGE (4–12% gel). Ubiquitylation reaction products were subjected to immunoblotting with an anti-FLAG antibody (Sigma, 1:10000), or anti-Parkin or anti-SUMO1 antibodies.

Experiments investigating the effect of ubiquitin^Phospho−Ser65^ and Ubl^Phospho−Ser65^ (isolated Parkin Ubl domain phosphorylated at Ser^65^) on Parkin activity, ubiquitylation reactions were performed in the absence of PINK1 in a final volume of 50 μl [50 mM Tris/HCl (pH 7.5), 5 mM MgCl_2_, 0.12 μM ubiquitin E1, 1 μM UbcH7, 2 μg of His_6_–SUMO-Miro1 and 2 mM ATP]. When the effects of ubiquitin^Phospho−Ser65^ were investigated, the total amount of ubiquitin used per assay was 25 μg; increasing concentrations of ubiquitin^Phospho−Ser65^ or non-phosphorylated ubiquitin were added as indicated and the final amount of ubiquitin was reached by the addition of FLAG–ubiquitin. When the effects of Ubl^Phospho−Ser65^ were investigated, 0.05 mM FLAG–ubiquitin was used and the Ubl^Phospho−Ser65^ or non-phosphorylated-Ubl were added as indicated. Ubiquitylation reactions were carried out at 30°C for 60 min, terminated by the addition of SDS sample buffer and subjected to immunoblotting as described above.

### *In vitro* E2-discharge assays

Wild-type or indicated mutants of Parkin (2 μg) were incubated with 1 μg of *E. coli*-expressed wild-type or kinase-inactive (D359A) MBP–TcPINK1 in a reaction volume of 15 μl [50 mM Hepes (pH 7.5), 10 mM magnesium acetate and 0.1 mM ATP]. Kinase assays were carried out at 30°C for 60 min. The E2-charging reaction was assembled in parallel in a 5 μl volume containing Ube1 (0.5 μg), UbcH7 (2 μg), 50 mM Hepes (pH 7.5) and 10 μM ubiquitin in the presence of 2 mM magnesium acetate and 0.2 mM ATP. After an initial incubation of 60 min at 30°C, the reactions were combined and allowed to continue for a further 15 min at 30°C. Reactions were terminated by the addition of 5 μl of SDS sample loading buffer and were subjected to SDS/PAGE (4–12% gel) analysis in the absence of any reducing agent. Gels were stained using InstantBlue.

During experiments investigating the effect of ubiquitin^Phospho−Ser65^ and Ubl^Phospho−Ser65^ on the Parkin-mediated E2 discharge, 1 μg of the indicated ubiquitin and Ubl species were combined with the E2-discharge reaction.

### Kinase assays

Reactions were set up in a volume of 25 μl, using 2 μg of wild-type or S65A ubiquitin mutants of Parkin and 1 μg of *E. coli*-expressed wild-type or kinase-inactive (D359A) MBP–TcPINK1, in 50 mM Tris/HCl (pH 7.5), 0.1 mM EGTA, 10 mM MgCl_2_, 2 mM DTT and 0.1 mM [γ-^32^P]ATP. Assays were incubated at 30°C with shaking at 1050 rev./min and terminated after 60 min by the addition of SDS sample loading buffer. The reaction mixtures were then resolved by SDS/PAGE (4–12% gel). Proteins were detected by Coomassie Blue staining and gels were imaged using an Epson scanner and dried completely using a gel dryer (Bio-Rad Laboratories). Incorporation of [γ-^32^P]ATP into substrates was analysed by autoradiography using Amersham Hyperfilm.

### Buffers for *E. coli* protein purification

For Parkin purification, the following buffers were used. Lysis buffer contained 50 mM Tris/HCl (pH 7.5), 150 mM NaCl, 1 mM EDTA, 1 mM EGTA, 5% (v/v) glycerol, 1% (v/v) Triton X-100, 0.1% 2-mercaptoethanol, 1 mM benzamidine and 0.1 mM PMSF. Wash buffer contained 50 mM Tris/HCl (pH 7.5), 500 mM NaCl, 0.1 mM EGTA, 5% (v/v) glycerol, 0.03% Brij-35, 0.1% 2-mercaptoethanol, 1 mM benzamidine and 0.1 mM PMSF. Equilibration buffer contained 50 mM Tris/HCl (pH 7.5), 150 mM NaCl, 0.1 mM EGTA, 5% (v/v) glycerol, 0.03% Brij-35, 0.1% 2-mercaptoethanol, 1 mM benzamidine and 0.1 mM PMSF. Elution buffer was equilibration buffer with the addition of 12 mM maltose. Storage buffer was equilibration buffer with the addition of 0.27 M sucrose and glycerol, PMSF and benzamidine were omitted.

### Protein purification from *E. coli*

Full-length wild-type or kinase-inactive TcPINK1 was expressed in *E. coli* as an MBP-fusion protein and purified as described previously [22]. Briefly, transformed BL21-CodonPlus (DE3)-RIL cells were grown at 37°C to a *D*_600_ of 0.3, then shifted to 16°C and induced with 250 μM IPTG at a *D*_600_ of 0.5. Cells were induced with 250 μM IPTG at a *D*_600_ of 0.6 and were further grown at 16°C for 16 h. Cells were pelleted at 3300 ***g*** (4°C for 10 min), and then lysed by sonication (45% amplitude, 10 s on/10 s off; 1 min 40 s total sonication time) in lysis buffer. Lysates were clarified by centrifugation at 30000 ***g*** for 30 min at 4°C followed by incubation with 1 ml of amylose resin/litre of culture for 1.5 h at 4°C. The resin was washed thoroughly in wash buffer followed by equilibration buffer, and proteins were then eluted. Proteins were dialysed overnight at 4°C into storage buffer, snap-frozen and stored at −80°C until use.

Wild-type and indicated mutant untagged Parkin (His_6_–SUMO-cleaved) was expressed and purified using a modified protocol [23]. BL21-CodonPlus (DE3)-RIL cells were transformed with His_6_–SUMO-tagged Parkin constructs, and overnight cultures were prepared and used to inoculate 12× 1 litre volumes of LB medium (containing 50 μg/ml carbenicillin and 0.25 mM ZnCl_2_). The cells were grown at 37°C until the *D*_600_ was 0.4 and the temperature was lowered to 16°C. At a *D*_600_ of 0.8 expression was induced with 25 μM IPTG. After overnight incubation the cells were collected and lysed in 75 mM Tris/HCl (pH 7.5), 500 mM NaCl, 0.2% Triton X-100, 25 mM imidazole, 0.5 mM TCEP [tris-(2-carboxyethyl)phosphine], 1 mM Pefabloc and 10 μg/ml leupeptin. After sonication (45% amplitude, 10 s on/10 s off; 1 min 40 s total sonication time) and removal of insoluble material, His_6_–SUMO–Parkin was purified using Ni-NTA (Ni^2+^-nitrilotriacetate)–Sepharose chromatography. The protein was collected by elution with 400 mM imidazole in 50 mM Tris/HCl (pH 8.2), 200 mM NaCl, 10% glycerol, 0.03% Brij-35 and 0.5 mM TCEP. This was dialysed twice against 50 mM Tris/HCl (pH 8.2), 200 mM NaCl, 10% glycerol and 0.5 mM TCEP in the presence of His_6_–SENP1-(415–643) at a ratio of 1 mg of His_6_–SENP1 per 5 mg of His_6_–SUMO–Parkin. The protease, the His_6_–SUMO tag and any uncleaved protein were removed by two subsequent incubations with Ni-NTA–Sepharose. The cleaved Parkin was further purified in 50 mM Tris/HCl (pH 8.2), 200 mM NaCl, 20% glycerol, 0.03% Brij-35 and 0.5 mM TCEP over a Superdex 200 column.

Wild-type His_6_–SUMO-Miro1-(1–592) was expressed in *E. coli*. Briefly, BL21-CodonPlus (DE3)-RIL transformed cells were grown at 37°C to a *D*_600_ of 0.4, then shifted to 15°C and induced with 10 μM IPTG at a *D*_600_ of 0.6. Cells were then grown at 15°C for a further 20 h. Cells were pelleted at 4200 ***g*** and then lysed by sonication (45% amplitude, 10 s on/10 s off; 1 min 40 s total sonication time) in lysis buffer. Lysates were clarified by centrifugation at 30000 ***g*** for 30 min at 4°C followed by incubation with cobalt resin at 4°C for 45 min. The resin was washed thoroughly in high-salt buffer, equilibrated in low-salt buffer, and the proteins were then eluted. The eluted Miro1 proteins were further purified by anion-exchange chromatography. Proteins were applied to a Mono-Q HR 5/5 column and chromatographed with a linear gradient of NaCl from 0 to 0.5 M. Fractions containing the purified Miro1 protein were then dialysed, snap-frozen in liquid nitrogen and stored at −70°C.

GST-fusion proteins were purified by similar methods in *E. coli* except that GST–MLK1 (mixed lineage kinase 1)-(132–413), GST–OTU1 [OTU (ovarian tumour) domain-containing protein 1], GST–ISG15 (interferon-induced 17 kDa protein) and GST–CDK2 (cyclin-dependent kinase 2)-(2–298)/cyclin A2-(171–432) were affinity-purified using GSH–Sepharose chromatography and for GST–CDK2 and GST–ISG15, the GST tag was cleaved with PreScission protease. His_6_–Aurora A-(2–403), His_6_–SUMO1 and His_6_–HOIL1 [haem-oxidized IRP2 (iron-regulatory protein 2) ubiquitin ligase 1] were purified by similar methods in *E. coli* except that recombinant His_6_-fusion protein was affinity-purified on Ni-NTA–agarose. Ubiquilin2 was purified by similar methods to that for the His_6_–SUMO fusion protein and then underwent His_6_–SUMO tag cleavage. Untagged Nedd8 (neural-precursor-cell-expressed developmentally down-regulated 8) was expressed as described previously [24]. His_6_–IKKε [IκB (inhibitor of nuclear factor κB) kinase ε]-(1–716), His_6_–IKKβ-(1–716), His_6_–PLK1 (Polo-like kinase 1)-(1–603), His_6_–NUAK1 (NUAK family SNF1-like kinase 1)-(2–660) and His_6_–GSK3β (glycogen synthase kinase-3β)-(2–420) were purified in a baculovirus expression vector system using Ni-NTA–agarose as described previously [25].

### Mapping the site on ubiquitin phosphorylated by TcPINK1

FLAG–ubiquitin (10 μg) was incubated with 10 μg of either wild-type MBP–TcPINK1 (residues 1–570) or kinase-inactive MBP–TcPINK1 (D359A) for 80 min at 30**°**C in 50 mM Tris/HCl (pH 7.5), 0.1 mM EGTA, 10 mM MgCl_2_, 0.15 2-mercaptoethanol and 0.1 mM [γ-^32^P]ATP (approximately 20000 c.p.m./pmol) in a total reaction volume of 50 μl. The reaction was terminated by the addition of SDS sample buffer with 10 mM DTT, boiled and subsequently alkylated with 50 mM iodoacetamide before samples were subjected to electrophoresis on a Bis-Tris 4–12% polyacrylamide gel, which was then stained with Colloidal Coomassie Blue (Invitrogen). Phosphorylated ubiquitin was digested with trypsin and 78% of the ^32^P radioactivity incorporated into ubiquitin was recovered from the gel bands. Peptides were chromatographed on a reverse-phase HPLC Vydac C_18_ column (catalogue number 218TP5215, Separations Group) equilibrated in 0.1% trifluoroacetic acid, and the column developed with a linear acetonitrile gradient at a flow rate of 0.2 ml/min before fractions (0.1 ml each) were collected and analysed for ^32^P radioactivity by Cerenkov counting. Isolated phosphopeptides were analysed by LC–MS/MS on a Thermo U3000 RSLC nano-LC system coupled to a Thermo LTQ-Orbitrap Velos mass spectrometer. The resultant data files were searched using Mascot (http://www.matrixscience.com) run on an in-house system against a database containing the ubiquitin sequence, with a 10 p.p.m. mass accuracy for precursor ions, a 0.6 Da tolerance for fragment ions, and allowing for Phospho (ST), Phospho (Y), Oxidation (M) and Dioxidation (M) as variable modifications. Individual MS/MS spectra were inspected using Xcalibur v2.2 software (Thermo Scientific). The site of phosphorylation of these ^32^P-labelled peptides was determined by solid-phase Edman degradation on a Shimadzu PPSQ33A sequencer of the peptide coupled to Sequelon-AA membrane (Applied Biosystems) as described previously [26].

### Purification of Ser^65^-phosphorylated ubiquitin and Ser^65^-phosphorylated Parkin Ubl domain (residues 1–76)

Bovine ubiquitin (23 μM; Sigma) was phosphorylated for 24 h with 3.7 μM MBP–TcPINK1 at 22°C in the presence of 100 μM ATP and 10 mM MgCl_2_. To replace ADP with ATP, the reaction was dialysed against Mg^2+^-ATP solution. Ubiquitin was filtered through a 30 kDa Vivaspin filter to remove MBP–TcPINK1, concentrated in a 3 kDa molecular-mass cut-off filter device, washed extensively with water and loaded on to a Mono Q column, which did not bind ubiquitin, but did bind phospho-ubiquitin. The latter was recovered by washing the column with 50 mM Tris/HCl (pH 7.5), which was sufficient to elute stoichiometrically phosphorylated ubiquitin. Similarly, Parkin Ubl domain (residues 1–76) was expressed as described previously [13] and was phosphorylated with MBP–TcPINK1, recovered by filtration and applied to a Mono Q column. Phospho-Parkin Ubl bound to the column and eluted with approximately 100 mM NaCl. At least 60% purity was achieved.

### MALDI analysis

MALDI–TOF was used to confirm and establish the ratios of phosphorylated compared with non-phosphorylated protein species. An aliquot of the reaction (2 μl, 400–600 fmol) was added to 2 μl of the matrix [2,5-dihydroxyacetophenone, 15 mg/ml in 80% ethanol and 20% 12 mg/ml ammonium citrate dibasic] and 2 μl of 2% (v/v) trifluoroacetic acid was added before spotting 0.5 μl of the sample on to an AnchorChip target (Bruker Daltonics). The analysis was performed manually in linear positive mode using an UltrafleXtreme (Bruker Daltonics) MALDI–TOF mass spectrometer. For external calibration, six average masses were used: insulin [*M*+H]^+^avg (*m*/*z* 5734.520), cytochrome *c* [*M*+2H]^2+^avg (*m*/*z* 6181.050), myoglobin [*M*+2H]^2+^avg (*m*/*z* 8476.660), ubiquitin I [*M*+H]^+^avg (*m*/*z* 8565.760) and cytochrome *c* [*M*+H]+avg (*m*/*z* 12360.970).

## RESULTS

### Overexpression of activated PINK1 in HEK-293 cells leads to phosphorylation of ubiquitin at Ser^65^

**Figure 1:**
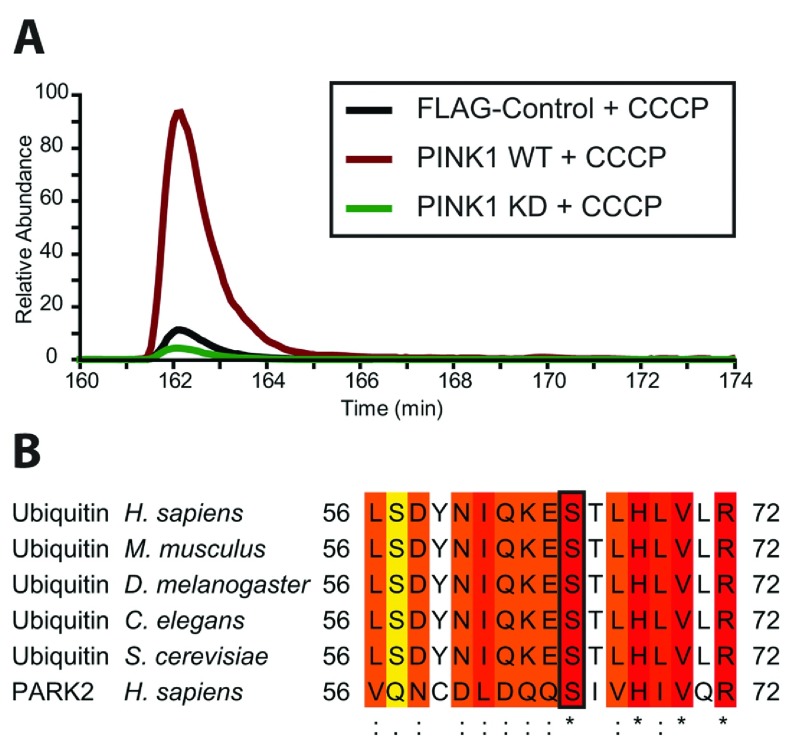
Identification of a highly conserved ubiquitin phospho-Ser^65^ peptide upon PINK1 stimulation by CCCP *in vivo* New highly conserved ubiquitin phospho-Ser^65^ peptide is up-regulated upon cell treatment with CCCP. Flp-In T-Rex HEK-293 cells stably expressing FLAG-empty, wild-type PINK1–FLAG or kinase-inactive PINK1–FLAG were grown in light, heavy and medium SILAC media respectively. Cells under each condition were stimulated with 10 μM CCCP for 3 h. Subsequently, membrane fractions were enriched by ultracentrifugation and solubilized in 1% RapiGest. Lysates from each of the three conditions were mixed at 1:1:1 and digested with trypsin before phosphopeptide enrichment by HILIC (hydrophilic-interaction LC) and TiO_2_, and analysis by MS. Data analysis was performed using MaxQuant. The experiment was performed using four replicates. (**A**) Representative extracted ion chromatograms representing the ubiquitin Ser^65^) phosphopeptide TLSDYNIQKEpSTLHLVLR in the three SILAC-labelled conditions. (**B**) Sequence alignment of residues around Ser^65^ in human Parkin and ubiquitin in a variety of organisms showing a high degree of conservation. *C. elegans*, *Caenorhabditis elegans*; *D. melanogaster*, *Drosophila melanogaster*; *H. sapiens*, *Homo sapiens*; *M. musculus*, *Mus musculus*; *S. cerevisiae*, *Saccharomyces cerevisiae*.

We have previously reported that PINK1 is activated in response to mitochondrial depolarization and that this leads to autophosphorylation of PINK1 at Thr^257^ as well as phosphorylation of Parkin at Ser^65^ *in vivo* [13]. To define whether PINK1 may have additional substrates that are critical for mediating PINK1 downstream signalling in response to mitochondrial depolarization, we undertook a SILAC-based quantitative phosphoproteomic screen in Flp-In T-Rex HEK-293 cells stably expressing FLAG empty, wild-type or kinase-inactive PINK1–FLAG. Cells were stimulated with 10 μM CCCP for 3 h to activate PINK1 and mitochondria-containing membrane-enriched fractions were made and solubilized in 1% RapiGest. Lysates were mixed from each cell condition at 1:1:1 before being subjected to tryptic digestion. Digested peptides were subjected to phospho-peptide enrichment and analysis by MS. The full analysis of the screen is available upon request (C. Kondapalli, B. Dill, J. Proctor, A. Kazlauskaite, M. Trost and M. Muqit, unpublished work). We strikingly identified a novel ubiquitin phosphopeptide (TLSDYNIQKEpSTLHLVLR; pS indicating that Ser^65^ on ubiquitin is phosphorylated) that was significantly enriched 14-fold in stimulated mitochondrial extracts of wild-type PINK1 compared with kinase-inactive PINK1 across all four biological replicates (Figure 1A, and Supplementary Figures S1 and S2 at http://www.biochemj.org/bj/460/bj4600127add.htm). Multiple sequence alignment of this region of ubiquitin revealed a high degree of conservation of this phosphorylation site across all species (Figure 1B) and the peptide also bears strong homology with the Parkin Ser^65^ peptide with a hydrophobic residue at the −4 position and histidine and valine residues at the +3 and +5 positions respectively (Figure 1B).

### Ubiquitin is a direct substrate of PINK1

**Figure 2:**
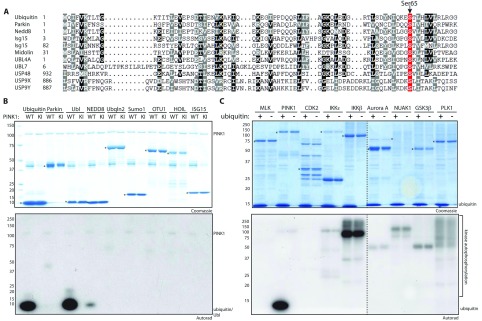
Ubiquitin is a direct substrate of TcPINK1 *in vitro* (**A**) Sequence alignment of residues around Ser^65^ in human ubiquitin and a range of ubiquitin-like modifiers and Ubl-domain-containing human proteins. (**B**) Ubiquitin and Parkin are specific substrates of TcPINK1. The indicated ubiquitin-like modifiers and Ubl-domain-containing proteins (1 μg) were incubated with wild-type (WT) or kinase-inactive (KI) TcPINK1 and Mg^2+^-[γ-^32^P]ATP for 60 min. Assays were terminated by the addition of SDS loading buffer and products were analysed by SDS/PAGE. Proteins were detected by Colloidal Coomassie Blue staining (top panel) and incorporation of [γ-^32^P]ATP was detected by autoradiography (bottom panel). All substrates were of human sequence and expressed in *E. coli*. Tags on the substrates used for this experiment were GST–OTU1, untagged Nedd8, untagged ISG15, His_6_–SUMO1-(1–97), ubiquilin2 (His_6_–SUMO tag cleaved off), His_6_–HOIL1 and USP4. Asterisks denote the correct substrate band. (**C**) TcPINK1 is a specific upstream kinase of ubiquitin. The indicated kinases (1 μg) were incubated with ubiquitin and Mg^2+^-[γ-^32^P]ATP for 60 min. Assays were terminated and analysed as described in (**B**). Except for PINK1, all kinases were of human sequence and expression tags used were GST–MLK1-(132–413), GST–CDK2-(2–298)/cyclin A2-(171–432); His_6_–IKKε-(1–716), His_6_–IKKβ-(1–716), His_6_–Aurora A-(2–403), His_6_–NUAK1-(2–660), His_6_–GSK3β-(2–420) and His_6_–PLK1-(1–603). Broken dividing lines indicate separate gels; asterisks denote the kinase band. The molecular mass in kDa is indicated.

We next investigated whether PINK1 could directly phosphorylate ubiquitin and other ubiquitin-like modifiers or proteins that contain a Ubl domain. Multiple sequence alignment identified additional human proteins that contain a phosphorylatable serine residue at the equivalent position to Parkin and ubiquitin (Figure 2A). These include ubiquitin-like modifiers, Nedd8 and ISG15 (both domains), and Ubl-domain-containing proteins including MIDN (midnolin), UBL4A, UBL7, USP48, USP9X and USP9Y. We expressed a subset of ubiquitin-like modifiers and Ubl-domain containing proteins with (ubiquitin, ISG15, Nedd8, Parkin, Parkin Ubl domain) or without (OTU1, HOIL, ubiquilin2, SUMO1) a predicted Ser^65^ residue and tested these in phosphorylation assays with catalytically active recombinant wild-type or kinase-inactive TcPINK1. Strikingly, ubiquitin was phosphorylated directly by wild-type TcPINK1 to a degree similar to the isolated Ubl domain of Parkin (Ubl) and greater than full-length Parkin (Figure 2B). No phosphorylation of these proteins was seen with kinase-inactive TcPINK1. We also observed phosphorylation of Nedd8, although this was significantly less than that observed for ubiquitin (Figure 2B).

To investigate the specificity of ubiquitin phosphorylation by PINK1, we compared the ability of eight other protein kinases (MLK, CDK2, IKKε, IKKβ, Aurora kinase A, NUAK1, GSK3β and PLK1) to phosphorylate ubiquitin. Using equimolar amounts of ubiquitin we only observed significant phosphorylation of ubiquitin by TcPINK1 and not any other kinase tested (Figure 2C).

### PINK1 phosphorylates ubiquitin at Ser^65^ *in vitro*

**Figure 3:**
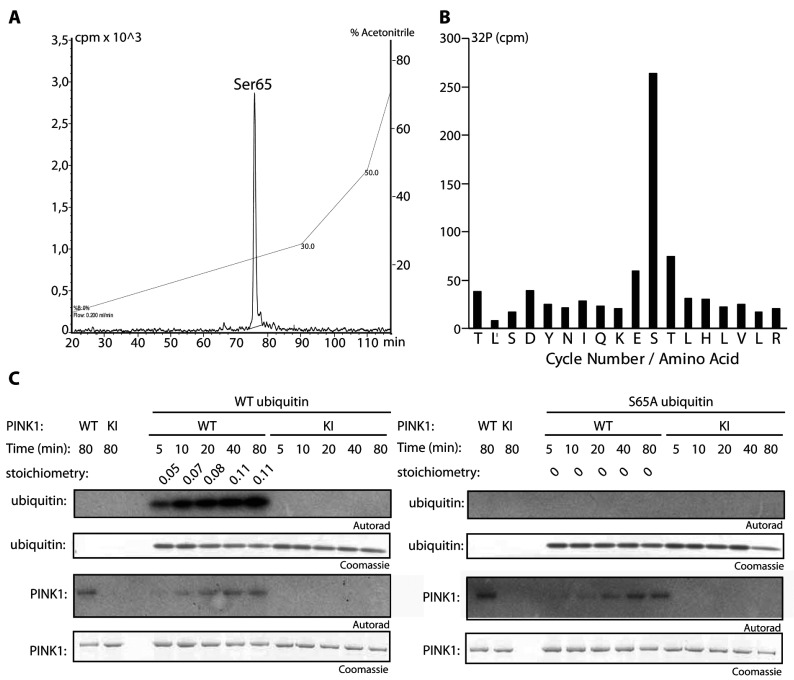
TcPINK1 phosphorylates ubiquitin at Ser^65^ (**A**) Mapping of phosphopeptide on ubiquitin after phosphorylation by TcPINK1 *in vitro*. Ubiquitin (10 μg) was incubated with 10 μg of either wild-type TcPINK1 or kinase-inactive TcPINK1 (D359A) in the presence of Mg^2+^-[γ-^32^P]ATP for 80 min. Assays were terminated by the addition of SDS loading buffer and products were separated by SDS/PAGE. Proteins were detected by Colloidal Coomassie Blue staining and phosphorylated ubiquitin was digested with trypsin. Peptides were chromatographed on a reverse-phase HPLC Vydac C_18_ column equilibrated in 0.1% trifluoroacetic acid and the column developed with a linear acetonitrile gradient at a flow rate of 0.2 ml/min and fractions (0.1 ml each) were collected and analysed for ^32^P radioactivity by Cerenkov counting. One major ^32^P-labelled peak was identified following incubation with wild-type TcPINK1, whereas no peaks were identified following incubation with kinase-inactive TcPINK1 (results not shown). (**B**) The phosphopeptide identified in (**A**) was analysed by solid-phase Edman sequencing and MS. The amino acid sequence deduced from the single phosphopeptide seen in the LC–MS/MS analysis is shown using the single-letter amino acid code. (**C**) The S65A mutation abolishes ubiquitin phosphorylation by TcPINK1. Wild-type or S65A mutant ubiquitin (1 μg) was incubated in the presence of wild-type or kinase-inactive TcPINK1 (1 μg) and Mg^2+^-[γ-^32^P]ATP for the times indicated and assays were terminated by the addition of SDS loading buffer. Samples were subjected to SDS/PAGE and proteins detected by Colloidal Coomassie Blue staining (bottom panels) and incorporation of [γ-^32^P]ATP was detected by autoradiography (top panels). Cerenkov counting was used to calculate the stoichiometry of ubiquitin phosphorylation indicated above autoradiographs as mol of [γ-^32^P]ATP incorporated/mol of ubiquitin. KI, kinase-inactive; WT, wild-type.

To determine the site of ubiquitin phosphorylation by wild-type TcPINK1, tryptic digests of ^32^P-labelled ubiquitin were analysed by chromatography on a C_18_ column and one major ^32^P-labelled phosphopeptide was observed (Figure 3A). A combination of solid-phase Edman sequencing and MS revealed a peptide phosphorylated at Ser^65^ (Figure 3B). We next undertook a time-course analysis of ubiquitin phosphorylation by TcPINK1 and observed robust time-dependent phosphorylation of ubiquitin by wild-type TcPINK1 that was not observed by incubation of kinase-inactive TcPINK1 (Figure 3C, left-hand panel). The maximal stoichiometry of ubiquitin phosphorylation by TcPINK1 under our assay conditions was ~0.11 mol of phosphate/mol of protein (Figure 3C, left-hand panel). Importantly, mutation of Ser^65^ to alanine abolished phosphorylation of ubiquitin by wild-type TcPINK1 confirming that this residue is the major site of PINK1 phosphorylation (Figure 3C, right-hand panel).

### PINK1-dependent phosphorylation of ubiquitin activates ΔUbl-Parkin E3 ligase activity

**Figure 4:**
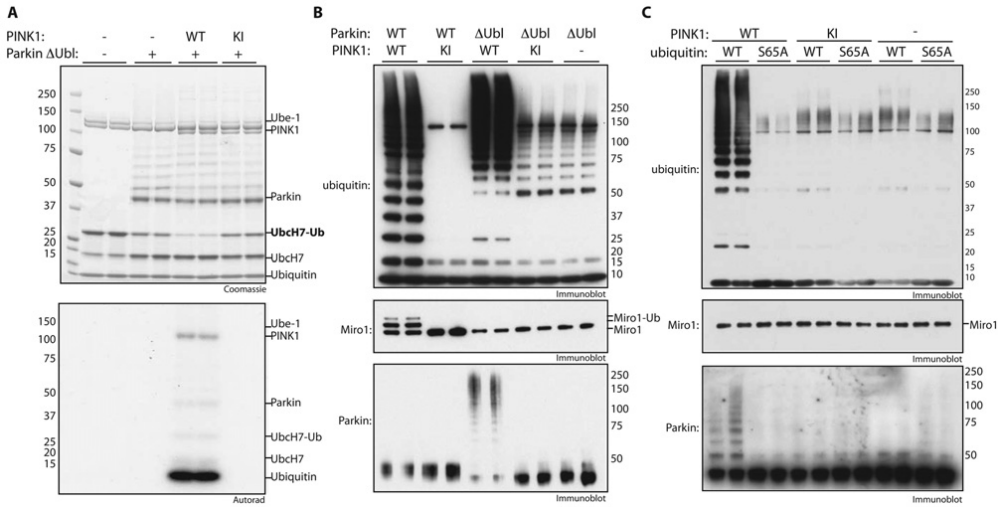
PINK1-dependent phosphorylation of ubiquitin at Ser^65^ leads to increased activity of ΔUbl-Parkin (**A**) MBP–TcPINK1 enhances ΔUbl-Parkin-mediated ubiquitin discharge from UbcH7. ΔUbl-Parkin was phosphorylated using wild-type (WT), kinase-inactive (KI) or no MBP–TcPINK1 in the presence of Mg^2+^-[γ-^32^P]ATP. An E2-discharge assay was established by incubation of this mixture with 2 μg of UbcH7 that had been pre-incubated with E1 and FLAG–ubiquitin in the presence of ATP for 60 min. Reactions were allowed to continue for 15 min and were stopped using SDS loading buffer in the absence of reducing agent. Samples were analysed as described in the Materials and methods section. Protein phosphorylation was monitored by autoradiography (bottom panel). (**B**) ΔUbl-Parkin activity is increased by wild-type TcPINK1. A 2 μg amount of full-length (WT) or ΔUbl-Parkin was incubated with 1 μg of wild-type (WT), kinase-inactive (KI) or no TcPINK1 in a kinase reaction for 60 min. The ubiquitylation reactions were then initiated by the addition of ubiquitylation assay components (E1, UbcH7 and FLAG–ubiquitin) and 2 μg of His_6_–SUMO-Miro1. Reactions were terminated after 60 min by the addition of SDS loading buffer and products were analysed by SDS/PAGE. Miro1, ubiquitin (Ub) and Parkin were detected using anti-SUMO, anti-FLAG and anti-Parkin antibodies respectively. (**C**) Activation of ΔUbl-Parkin by TcPINK1 is abolished by S65A ubiquitin. ΔUbl-Parkin was incubated in the presence or absence of wild-type (WT) or kinase-inactive (KI) PINK1. The ubiquitylation reactions were then initiated by the addition of ubiquitylation assay components including 0.04 mM wild-type (WT) or S65A His_6_–FLAG–ubiquitin. Reactions were terminated after 60 min by the addition of SDS loading buffer and products were analysed by SDS/PAGE. Miro1, ubiquitin and Parkin were detected using anti-SUMO, anti-FLAG and anti-Parkin antibodies respectively. The molecular mass in kDa is indicated.

As outlined in the introduction, we recently reported that incubating Parkin in the presence of wild-type PINK1, ubiquitin and Mg^2+^-ATP resulted in phosphorylation of Parkin at Ser^65^, which was accompanied by a marked stimulation of Parkin E3 ligase activity [13,27]. In the light of the finding that PINK1 phosphorylates ubiquitin at Ser^65^, we decided to explore whether the ability of PINK1 to activate Parkin was dependent on the presence of phosphorylated ubiquitin. We initially studied a fragment of Parkin that lacks the Ubl domain and hence Ser^65^ (residues 80–465; ΔUbl-Parkin) and which therefore cannot be directly phosphorylated by PINK1. We and other groups [23,27,28] have previously demonstrated that this fragment exhibits enhanced basal E3 ligase activity compared with full-length non-phosphorylated Parkin. We investigated whether addition of PINK1 in the presence of Mg^2+^-ATP and ubiquitin (which would lead to the production of Ser^65^-phosphorylated ubiquitin) could lead to enhanced activation of ΔUbl-Parkin using a recently established E2-ubiquitin discharge assay in which the hydrolysis of UbcH7 conjugated to ubiquitin is monitored on a Coomassie Blue-stained gel [27]. Using this assay, we observed that addition of ΔUbl-Parkin alone in the absence of PINK1 only caused mild E2-ubiquitin discharge, consistent with its moderate constitutive activity (Figure 4A, lanes 3 and 4). However, upon addition of wild-type, but not kinase-inactive, TcPINK1, we observed striking discharge of ubiquitin from UbcH7 (Figure 4A, lanes 5 and 6). The E2-discharge assay was conducted using 0.1 mM [γ-^32^P]ATP and autoradiographs revealed significant ubiquitin phosphorylation and TcPINK1 autophosphorylation during the reaction; no significant phosphorylation of any other component was observed (Figure 4A, bottom panel).

In addition to the E2-discharge assay, in our previous work we also assessed Parkin E3 ligase activity by monitoring the formation of free polyubiquitin chains, as well as the multi-mono-ubiquitylation of the substrate Miro1 [27]. In agreement with previous data, we observed marked activation of full-length Parkin E3 ligase activity when incubated with wild-type, but not kinase-inactive, PINK1 in the presence of ubiquitin and Mg^2+^-ATP as judged by the formation of free polyubiquitin chains and Miro1 multi-mono-ubiquitylation (Figure 4B, lanes 1 and 2). Moreover, consistent with previous work [23,27,28], ΔUbl-Parkin in the absence of PINK1 displayed significant basal polyubiquitylation activity (Figure 4B, lanes 9 and 10). Interestingly, upon incubation with wild-type, but not kinase-inactive, TcPINK1, we observed further substantial activation of ΔUbl-Parkin as judged by high-molecular-mass polyubiquitin chain formation and auto-ubiquitylation of Parkin (Figure 4B, lanes 5 and 6). It should be noted that, in contrast with full-length Parkin, we found that ΔUbl-Parkin did not catalyse Miro1 multi-mono-ubiquitylation when incubated with wild-type PINK1 (Figure 4B), indicating that the Ubl domain may be required for optimal substrate ubiquitylation.

We next sought to determine whether activation of ΔUbl-Parkin mediated by wild-type PINK1 was dependent on phosphorylation of ubiquitin at Ser^65^. We therefore repeated the ΔUbl-Parkin ubiquitylation assays described above in the presence of either wild-type or S65A mutant ubiquitin. Strikingly we observed a dramatic reduction in the activity of ΔUbl-Parkin pre-incubated with wild-type PINK1 when using S65A mutant ubiquitin in the ubiquitylation assay (Figure 4C, lanes 3 and 4) compared with using wild-type ubiquitin (Figure 4C, lanes 1 and 2).

### Evidence for dual requirement of PINK1-dependent phosphorylation of Parkin at Ser^65^ and ubiquitin at Ser^65^ in mediating optimal activation of full-length Parkin E3 ligase activity

**Figure 5:**
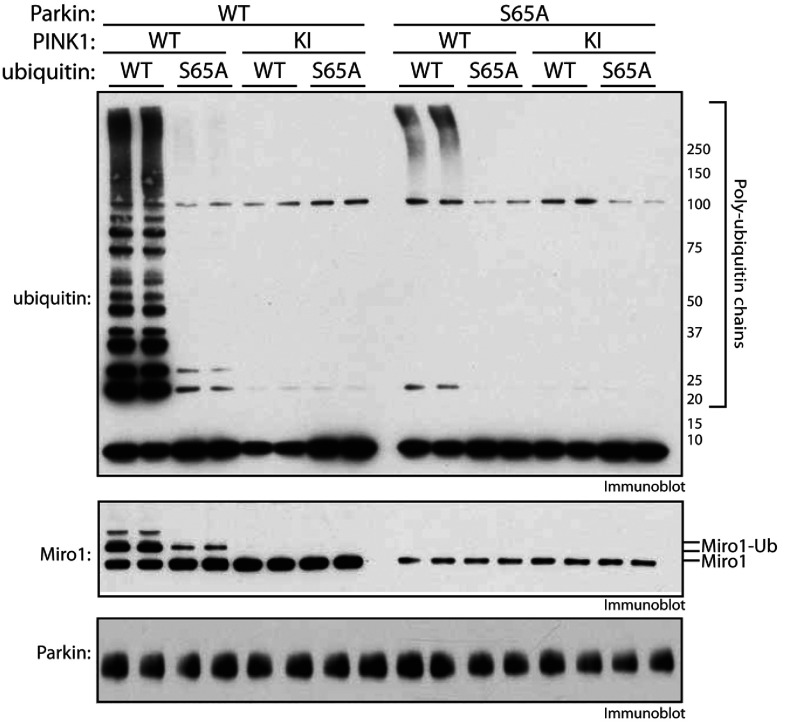
Phosphorylation of Parkin at Ser^65^ and ubiquitin at Ser^65^ are necessary for full activation by PINK1 Full-length (WT) and S65A Parkin were incubated in the presence or absence of wild-type (WT) or kinase-inactive (KI) PINK1 in a kinase reaction. The ubiquitylation reactions were then initiated by the addition of ubiquitylation assay components including 0.04 mM wild-type (WT) or S65A His_6_–FLAG–ubiquitin. Miro1, ubiquitin and Parkin were detected using anti-SUMO, anti-FLAG and anti-Parkin antibodies respectively. Miro1-Ub, ubiquitylated Miro1. The molecular mass in kDa is indicated.

We next investigated the role for PINK1-dependent phosphorylation of ubiquitin at Ser^65^ in the activation of full-length Parkin following TcPINK1 phosphorylation. Upon incubation of full-length Parkin with wild-type TcPINK1, ubiquitin and Mg^2+^-ATP we observed maximal Parkin ubiquitylation activity as judged by the generation of free polyubiquitin chains and multi-mono-ubiquitylation of Miro1 (Figure 5, lanes 1 and 2). The absolute dependence of Parkin on wild-type PINK1 for activation is confirmed by the absence of activity when Parkin is pre-incubated with kinase-inactive PINK1 (Figure 5, lanes 5 and 6). In the presence of a S65A mutant of ubiquitin, we observed a substantial decrease in Parkin E3 ligase activity; however, it was not completely abolished as judged by Miro1 ubiquitylation (Figure 5, lanes 3 and 4) as opposed to Parkin that was pre-incubated with kinase-inactive PINK1 (Figure 4C, lanes 7 and 8). Moreover, as we have previously reported [13,27], the Parkin S65A mutant led to near complete loss of Parkin activity as judged by loss of both Miro1 substrate ubiquitylation and free chain formation. We did observe high-molecular-mass polyubiquitylation (Figure 5, lanes 9 and 10) indicating residual activity. That either the S65A ubiquitin mutant or the Parkin S65A mutant alone can substantially reduce, but not abolish, Parkin E3 ligase activity indicates that both PINK1-dependent phosphorylation of Parkin at Ser^65^ and ubiquitin at Ser^65^ are required for mediating optimal activation of Parkin E3 ligase activity. Conversely we only observed complete loss of Parkin activity upon incubation of both S65A ubiquitin with S65A Parkin pre-incubated with wild-type TcPINK1 (Figure 5, lanes 11 and 12), similar to incubation of S65A Parkin pre-incubated with kinase-inactive TcPINK1 (Figure 5, lanes 13–16).

### Purified Ser^65^-phosphorylated ubiquitin and Ser^65^-phosphorylated Ubl domain of Parkin can directly and differentially activate Parkin E3 ligase activity

**Figure 6:**
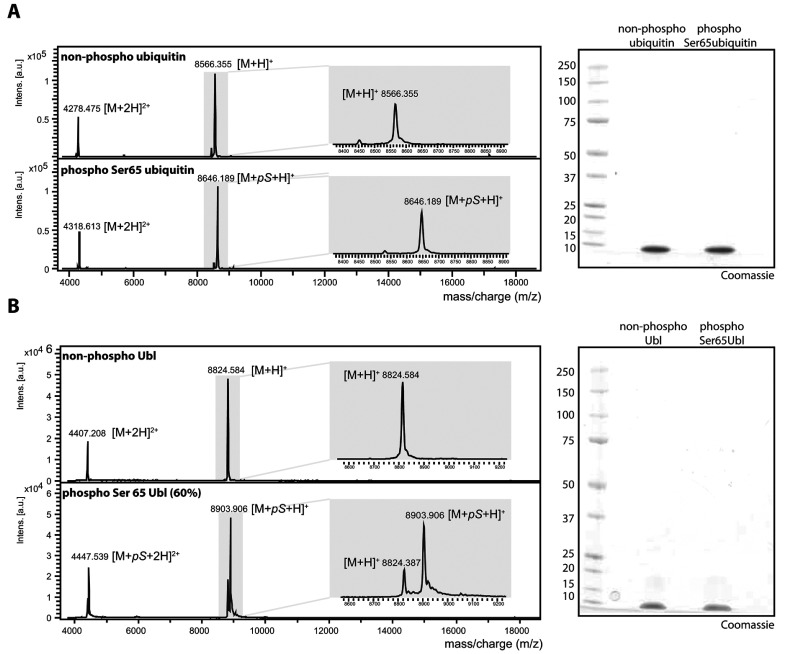
Quality control of phosphorylated Ubl (Parkin residues 1–76) and phosphorylated ubiquitin (**A**) MALDI–TOF spectra of non-phospho-ubiquitin (top panel) and phospho-Ser^65^ ubiquitin (bottom panel) after incubation with MBP–PINK1 after separation on a Mono Q column. (**B**) MALDI–TOF spectra of non-phosphorylated Ubl (Parkin residues 1–76) (top panel) and mixed Ubl species (~60% phosphorylated and ~40% non-phosphorylated) (bottom panel). (**A** and **B**) In total, 2 μg of the non-phospho-ubiquitin and phospho-ubiquitin (**A**) or non-phospho-Ubl and phospho-Ser^65^ Ubl (**B**) were resolved by SDS/PAGE, followed by staining with Colloidal Coomassie Blue for quality control.

To provide stronger evidence that ubiquitin phosphorylated at Ser^65^ can directly activate Parkin E3 ligase activity, we next investigated whether addition of purified Ser^65^-phosphorylated ubiquitin (ubiquitin^Phospho−Ser65^) and/or the Ser^65^-phosphorylated isolated Ubl domain of Parkin (residues 1–76) (Ubl^Phospho−Ser65^) could directly activate Parkin E3 ligase activity without the presence of wild-type PINK1. Milligram amounts of ubiquitin and the Ubl domain of Parkin (His_6_–SUMO tag cleaved) were phosphorylated by MBP–TcPINK1. MBP–TcPINK1 was then removed using a Centricon centrifugal high-molecular-mass filter and the reaction mixture was subjected to ion-exchange chromatography. We were able to successfully isolate 100% pure ubiquitin^Phospho−Ser65^ and non-phosphorylated ubiquitin to homogeneity and confirmed their identity and purity by MS (Figure 6A). We were able to purify at least 60% pure Ubl^Phospho−Ser65^ (Figure 6B). Importantly, they were free of TcPINK1.

**Figure 7:**
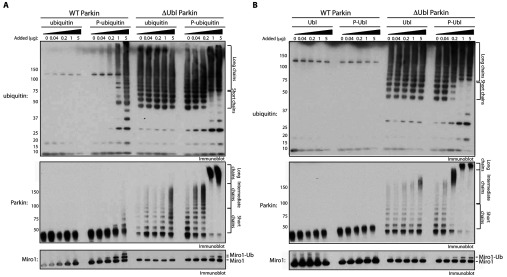
Full-length wild-type Parkin is activated by ubiquitin^Phospho−Ser65^, and ΔUbl-Parkin is activated by ubiquitin^Phospho−Ser65^ and Ubl^Phospho−Ser65^ (**A**) Full-length wild-type (WT) and ΔUbl-Parkin are activated by ubiquitin^Phospho−Ser65^. A 2 μg amount of full-length (WT), S65A or ΔUbl-Parkin was incubated with the ubiquitylation assay components (E1 and UbcH7) in the presence of 0.05 mM ubiquitin. The ubiquitin used in these assays was a mixture of FLAG–ubiquitin and untagged phospho- or non-phospho-ubiquitin as indicated (total of 25 μg per reaction). Reactions were terminated after 60 min by the addition of SDS loading buffer and products were analysed by SDS/PAGE. Miro1, ubiquitin and Parkin were detected using anti-SUMO, anti-FLAG and anti-Parkin antibodies respectively. Formation of short, intermediate and long polyubiquitylated species is indicated by square brackets. P-ubiquitin, ubiquitin^Phospho−Ser65^. (**B**) Ubl^Phospho−Ser65^ activates ΔUbl-Parkin, but not full-length Parkin. Reactions were carried out as described in (**A**), but the 0.05 mM FLAG–ubiquitin was used in all reactions and phospho- or non-phospho-Ubl domain (Parkin residues 1–76) was added as indicated. P-Ubl, Ubl^Phospho−Ser65^. The molecular mass in kDa is indicated.

We next assessed whether ubiquitin^Phospho−Ser65^ and/or Ubl^Phospho−Ser65^ could directly influence the activity of Parkin. We therefore performed Miro1 substrate ubiquitylation assays of either full-length wild-type or ΔUbl-Parkin (~0.8 μM) in the absence of TcPINK1 and investigated the effect of adding increasing amounts of ubiquitin^Phospho−Ser65^ or non-phosphorylated ubiquitin (0.04, 0.2, 1 and 5 μg). In the absence of ubiquitin^Phospho−Ser65^ we observed no activity of full-length Parkin (Figure 7A, lanes 1 and 6) and moderate basal activity of ΔUbl-Parkin (Figure 7A, lanes 11 and 16). Upon addition of increasing amounts of ubiquitin^Phospho−Ser65^ we observed striking activation of both full-length Parkin (Figure 7A, lanes 7–10) and ΔUbl-Parkin (Figure 7A, lanes 17–20) as judged by the formation of polyubiquitin chains, increased auto-ubiquitylation and Miro1 ubiquitylation. Importantly, we observed no activation of full-length Parkin (Figure 7A, lanes 2–5) or ΔUbl-Parkin (Figure 7A, lanes 12–15) following the addition of an equivalent amount of non-phosphorylated ubiquitin.

Interestingly, we observed differential sensitivity of full-length Parkin and ΔUbl-Parkin to the effect of ubiquitin^Phospho−Ser65^. Addition of 0.04 μg (~0.1 μM) of ubiquitin^Phospho−Ser65^ led to activation of ΔUbl-Parkin (Figure 7A, lane 17), but this amount did not have any significant effect on full-length Parkin (Figure 7A, lane 7). Furthermore, addition of the increasing amounts of ubiquitin^Phospho−Ser65^ led to a striking differential profile in the nature of the poly-auto-ubiquitylation catalysed by ΔUbl-Parkin with intermediate-sized chains formed on addition 0.04 μg or 0.2 μg of ubiquitin^Phospho−Ser65^ (Figure 7A, lanes 17 and 18) and high-molecular-mass long chains on addition of 1 (~2.5 μM) or 5 (~12 μM) μg of ubiquitin^Phospho−Ser65^ (Figure 7A, lanes 19 and 20). Moreover, we also observed that addition of molar excess amounts of ubiquitin^Phospho−Ser65^ (relative to ΔUbl-Parkin) enabled ΔUbl-Parkin to detectably catalyse mono-ubiquitylation of Miro1 (Figure 7A, lanes 19 and 20).

We then investigated the role that Ubl^Phospho−Ser65^ had on Parkin activity. Interestingly, addition of increasing amounts of Ubl^Phospho−Ser65^ (0.04, 0.2, 1 and 5 μg) had no significant effect on the activation of full-length Parkin (Figure 7B, lanes 7–10). In contrast, addition of Ubl^Phospho−Ser65^ induced a striking increase in activity of ΔUbl-Parkin as judged by polyubiquitin chain formation, increased auto-ubiquitylation and Miro1 mono-ubiquitylation (Figure 7B, lanes 17–20) similar to the effects induced by ubiquitin^Phospho−Ser65^.

### Purified Ser^65^-phosphorylated ubiquitin and Ser^65^-phosphorylated Ubl domain of Parkin can directly and differentially stimulate Parkin to discharge ubiquitin from UbcH7-loaded E2 ligase

**Figure 8:**
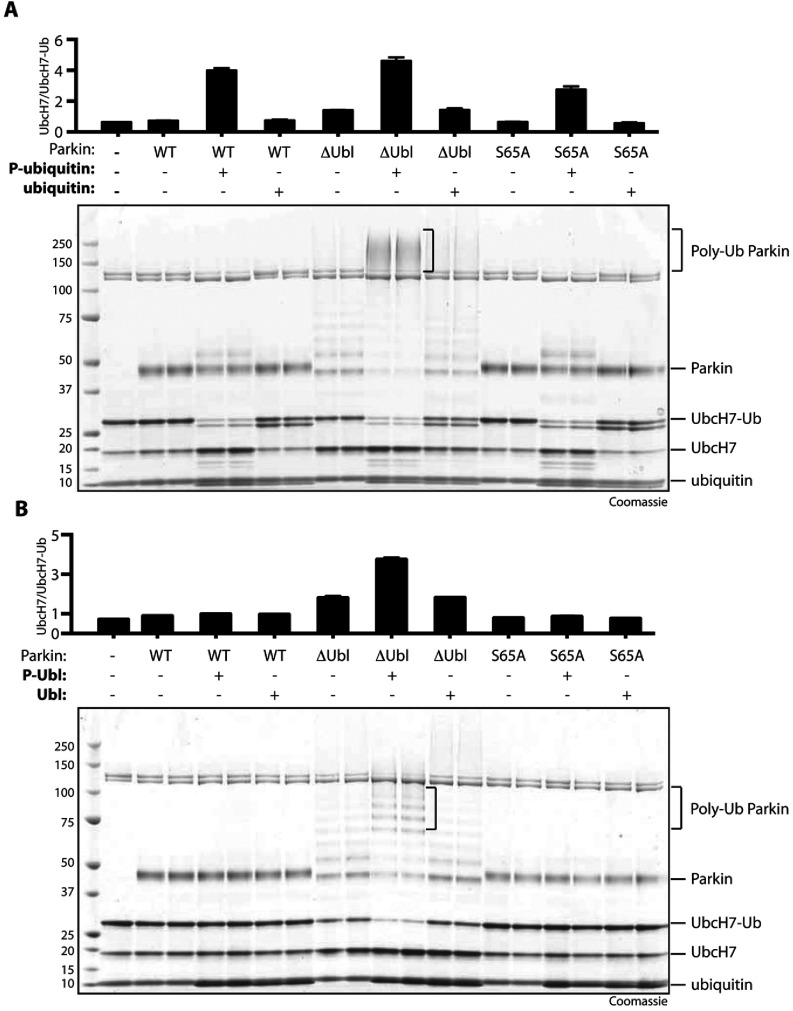
Differential effects of ubiquitin^Phospho−Ser65^ and Ubl^Phospho−Ser65^ on ubiquitin discharge by full-length wild-type, S65A and ΔUbl-Parkin (**A**) Ubiquitin^Phospho−Ser65^ leads to activation of full-length wild-type, S65A and ΔUbl-Parkin and increased ubiquitin discharge. An E2-discharge assay was established by incubation of full-length (WT), S65A or ΔUbl-Parkin in the presence or absence of phospho- or non-phospho-ubiquitin (1 μg) as indicated with 2 μg of UbcH7 that had been pre-incubated with E1 and FLAG–ubiquitin in the presence of Mg^2+^-ATP for 60 min. Reactions were allowed to continue for 15 min and were stopped using SDS loading buffer in the absence of reducing agent. The reaction products were resolved using SDS/PAGE and the proteins were visualised by Colloidal Coomassie Blue staining. (**B**) Ubl^Phospho−Ser65^ leads to activation and increased ubiquitin discharge by ΔUbl-Parkin, but does not affect the full-length wild-type and S65A Parkin. The assays were carried out as described above, but phospho- and non-phospho-Ubl were added to the reactions as indicated. P-ubiquitin, ubiquitin^Phospho−Ser65^; P-Ubl, Ubl^Phospho−Ser65^. The molecular mass in kDa is indicated.

We next evaluated the ability of purified ubiquitin^Phospho−Ser65^ or Ubl^Phospho−Ser65^ to stimulate Parkin to discharge ubiquitin from UbcH7-loaded E2 ligase in the absence of TcPINK1 as another read-out of Parkin E3 ligase activity. In these studies we investigated the ability of a fixed amount of ubiquitin^Phospho−Ser65^ or non-phosphorylated ubiquitin (1 μg) to stimulate wild-type full-length Parkin, a S65A mutant of full-length Parkin, as well as ΔUbl-Parkin to discharge ubiquitin from the loaded UbcH7. Addition of full-length Parkin alone to the ubiquitin-loaded E2 reaction did not promote E2-ubiquitin discharge (Figure 8A, lanes 2 and 3). However, addition of ubiquitin^Phospho−Ser65^ led to striking and maximal E2-ubiquitin discharge (Figure 8A, lanes 4 and 5) that was not observed following addition of non-phosphorylated ubiquitin (Figure 8A, lanes 6 and 7). Addition of ΔUbl-Parkin alone promoted slight E2-ubiquitin discharge (Figure 8A, lanes 8 and 9), but addition of ubiquitin^Phospho−Ser65^, triggered significantly increased E2-ubiquitin discharge (Figure 8A, lanes 10 and 11) that was not seen with the addition of non-phosphorylated ubiquitin (Figure 8A, lanes 12 and 13).

Interestingly, analysis of the Parkin S65A mutant revealed similar results to those of wild-type Parkin: we observed maximal discharge of E2-ubiquitin by S65A Parkin on addition of ubiquitin^Phospho−Ser65^ (Figure 8A, lanes 16 and 17), but not with non-phosphorylated ubiquitin (Figure 8A, lanes 18 and 19), indicating that, under the assay conditions used, ubiquitin^Phospho−Ser65^ alone is sufficient to activate even the S65A Parkin mutant.

We next tested the ability of Ubl^Phospho−Ser65^ to stimulate Parkin to discharge ubiquitin from UbcH7-loaded E2 ligase. Consistent with our analysis of the effect of Ubl^Phospho−Ser65^ on Parkin ubiquitylation (Figure 7B), we observed that Ubl^Phospho−Ser65^ (~5 μM) failed to stimulate full-length Parkin to discharge ubiquitin (Figure 8B, lanes 4 and 5). In contrast, Ubl^Phospho−Ser65^ markedly stimulated ΔUbl-Parkin to promote E2-ubiquitin discharge (Figure 8B, lanes 10 and 11), compared with ΔUbl-Parkin alone (Figure 8B, lanes 8 and 9) or ΔUbl-Parkin in combination with non-phosphorylated Ubl (Figure 8B, lanes 12 and 13). Similar to full-length wild-type Parkin, we observed that Ubl^Phospho−Ser65^ could not stimulate the S65A Parkin mutant to promote E2-ubiquitin discharge (Figure 8B, lanes 16 and 17).

## DISCUSSION

**Figure 9:**
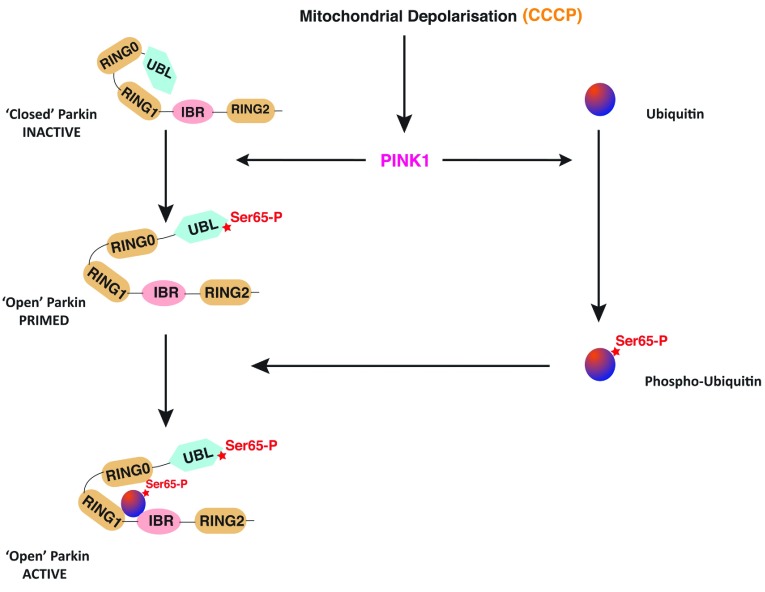
Model of Parkin activation by PINK1 and phospho-ubiquitin Under basal conditions Parkin exists in a closed inactive conformation, in part mediated by Ubl-mediated autoinhibition. Upon mitochondrial depolarization, induced by the uncoupler CCCP, PINK1 becomes activated and phosphorylates Parkin and ubiquitin at Ser^65^. Phosphorylation of Parkin relieves the Ubl-mediated autoinhibition and primes Parkin for optimal binding by phospho-Ser^65^-ubiquitin and activation of Parkin E3 ligase activity.

How Parkin is activated has been a major question since the discovery that Parkin is autoinhibited via interaction between the N-terminal Ubl domain and the C-terminal portion of the protein [23]. We have previously reported that PINK1-dependent phosphorylation of Parkin Ser^65^ that lies within the Ubl domain stimulates Parkin E3 ligase activity [13]. Recent high-resolution crystal structures of Parkin that were mainly solved without the Ubl domain have not provided much insight into how Parkin is activated by PINK1 [14–16]. In the present paper we describe a crucial discovery that PINK1-dependent phosphorylation of ubiquitin at Ser^65^ plays a critical role in mediating Parkin activation. Our data indicate that ubiquitin^Phospho−Ser65^ is a direct and potent activator of Parkin (Figures 7 and 8). Our studies with purified ubiquitin^Phospho−Ser65^ also demonstrate that the ΔUbl-Parkin requires smaller amounts of ubiquitin^Phospho−Ser65^ compared with full-length Parkin for activation (Figure 7). This might be consistent with a two-step activation mechanism of Parkin in which an initial phosphorylation of Parkin Ser^65^ by PINK1 within the Ubl domain would lead to disruption of the interaction of the Ubl domain and C-terminus of Parkin that could then prime Parkin for optimal binding and activation by ubiquitin^Phospho−Ser65^ (Figure 9).

Previous work has revealed that the Ubl domain of Parkin interacts with high affinity with the C-terminal catalytic domain [23]. Ser^65^ on the Ubl domain lies within the fifth β-strand and phosphorylation could perturb the β-strand formation around this region [29–32]. This could reduce affinity of the Ser^65^-phosphorylated Ubl domain for the catalytic region of Parkin, thereby opening up the Parkin structure and making it more readily activated by ubiquitin^Phospho−Ser65^. Such a priming model is well established for many protein kinases, e.g. for AGC kinases such as Akt1. Here Akt1 binds to the PtdIns(3,4,5)*P*_3_ second messenger produced by activation of PI3K (phosphoinositide 3-kinase). This does not directly activate Akt, but instead induces a conformational change that exposes the activating Thr^308^, priming it for phosphorylation by the upstream 3-phosphoinositide-dependent kinase 1 [33]. To our knowledge the only report for a potential priming mechanism for an RING E3 ligases is for the CBL-B RING E3 ligase, in which phosphorylation of Tyr^363^ that lies in a linker region away from the canonical RING domain plays a critical priming step to increase CBL-B activity via improving the efficiency of ubiquitin transfer from the E2 [34]. To support our priming model, in future work, it would be interesting to test whether purified PINK1-induced Ser^65^-phosphorylated wild-type full-length Parkin (in which the PINK1 has been removed) exhibits enhanced sensitivity to the activating effect of ubiquitin^Phospho−Ser65^ compared with non-phosphorylated Parkin. We have also recently identified a missense mutant of Parkin, R33Q, that lies within the Ubl domain which enables it to be phosphorylated to a significantly greater degree than wild-type Parkin and this is associated with increased E3 ligase activity [27]. It would be interesting to determine whether its enhanced activity was due to increased sensitivity to ubiquitin^Phospho−Ser65^.

Our findings also predict that there will be a ubiquitin^Phospho−Ser65^-binding pocket within the catalytic core of Parkin that would induce a conformational change to activate Parkin following the ubiquitin^Phospho−Ser65^ interaction. Recent structural analysis of Parkin has predicted a putative phosphopeptide-binding pocket within the RING0 domain that is formed by three positively charged residues, Lys^161^, Arg^163^ and Lys^211^ [15]. In the future it will be important to undertake binding studies and activation assays to determine whether mutation of these residues disrupts the ability of ubiquitin^Phospho−Ser65^ to bind to Parkin and become activated by PINK1.

We also observed a differential effect of the Ubl^Phospho−Ser65^ in its ability to activate Parkin. In contrast with ubiquitin^Phospho−Ser65^, Ubl^Phospho−Ser65^ was unable to activate full-length Parkin. We cannot rule out the possibility that this observation is due to the Ubl^Phospho−Ser65^ protein used being only 60% phosphorylated compared with ubiquitin^Phospho−Ser65^ which was stoichiometrically phosphorylated. However, we believe that this is unlikely since the Ubl^Phospho−Ser65^ was still capable of activating ΔUbl-Parkin to a similar degree as ubiquitin^Phospho−Ser65^. An alternative explanation may be that the binding pocket for Ubl^Phospho−Ser65^ is distinct from that of ubiquitin^Phospho−Ser65^ and not accessible due to steric hindrance imposed by the non-phosphorylated Ubl domain. We hypothesize that phosphorylation of Parkin at Ser^65^ would lead to release of this Ubl-mediated steric hindrance. This is supported by our observation that PINK1-induced phosphorylation of Parkin can still activate E3 ligase activity in the presence of S65A ubiquitin as judged by Miro1 ubiquitylation and this is abolished by the Parkin S65A mutant (Figure 5). It would be interesting to test this in future activation studies with of Ubl^Phospho−Ser65^ with phosphorylated Parkin. It would also be interesting to map and compare the region of interaction of Ubl^Phospho−Ser65^ and Parkin with that of ubiquitin^Phospho−Ser65^. Further characterization of the ubiquitin^Phospho−Ser65^/Ubl^Phospho−Ser65^ pocket on Parkin would be worthwhile as it might provide insights into how to generate small molecules that could bind to this site to activate Parkin. As loss-of-function mutations in PINK1 or Parkin cause PD, it would be fascinating to explore whether compounds that could bind to this site and activate Parkin would have therapeutic potential.

There is increasing evidence of a strong interplay between protein phosphorylation and protein ubiquitylation [35,36], but the discovery that PINK1 directly targets ubiquitin at Ser^65^ is perhaps the starkest example of how these two major systems of post-translational modifications converge. To our knowledge this is the first report of the identification of ubiquitin^Phospho−Ser65^. Previously large-scale phosphoproteomic screens have uncovered other phosphorylation sites of ubiquitin including Thr^7^ and Thr^12^ [37], Ser^57^ [38,39] and Tyr^59^ [40]; however, the functional consequences of phosphorylation of these sites on ubiquitin function is unknown, and neither is the identity of the upstream kinases for these sites known. Our discovery of ubiquitin^Phospho−Ser65^ raises many exciting future questions including whether it can be assembled in ubiquitin chains in a similar fashion to non-phosphorylated ubiquitin, whether E2 ligases can be charged with ubiquitin^Phospho−Ser65^, and whether other RING or RING-IBR-RING E3 ligases can use or be activated by ubiquitin^Phospho−Ser65^. Specifically it is interesting that Ser^65^ of ubiquitin lies close to Lys^63^ which forms a major ubiquitin linkage type. In future work it would be worth investigating whether phosphorylation at Ser^65^ of ubiquitin has an impact on its ability to form Lys^63^ chains and conversely whether dimeric or multimeric Lys^63^ chains composed of ubiquitin^Phospho−Ser65^ alters their sensitivity to Lys^63^-specific deubiquitinases such as AMSH-like protease [41].

Overall our studies have identified PINK1 as an upstream kinase of ubiquitin Ser^65^ and revealed a critical requirement of ubiquitin^Phospho−Ser65^ in mediating the activation of Parkin E3 ligase activity. Our work provides further mechanistic detail on the regulation of Parkin and suggests a dual mechanism in which both the Ser^65^-phosphorylated Ubl domain and ubiquitin^Phospho−Ser65^ are required for optimal Parkin activation. We also provide *in vivo* evidence that ubiquitin phosphorylation at Ser^65^ is regulated by PINK1 and it would be interesting to identify substrates *in vivo* that are modified by ubiquitin^Phospho−Ser65^ as these could represent physiological substrates of Parkin. Our findings also indicate that small molecules that mimic ubiquitin^Phospho−Ser65^ could be beneficial in activating Parkin as a potential therapy for PD patients.

## Online data

Supplementary data
