# Supplementary material for: Parkin is activated by PINK1-dependent phosphorylation of ubiquitin at Ser65
Source: Biochem J. 2014 Apr 25;460(Pt 1):127–39. doi: 10.1042/BJ20140334 (PMC4000136; doi:10.1042/BJ20140334)
Supplement: Supplementary data [file bj4600127add.pdf]

BJ Signal

Biochem. J. (2014) **460**, 127–139 (Printed in Great Britain) doi:10.1042/BJ20140334

online data

SUPPLEMENTARY ONLINE DATA

Parkin is activated by PINK1-dependent phosphorylation of ubiquitin at Ser[65]

Agne KAZLAUSKAITE*[1], Chandana KONDAPALLI*, Robert GOURLAY*, David G. CAMPBELL*, Maria Stella RITORTO*, Kay HOFMANN†, Dario R. ALESSI*, Axel KNEBEL*, Matthias TROST* and Miratul M. K. MUQIT*‡[1]

*MRC Protein Phosphorylation and Ubiquitylation Unit, University of Dundee, Dundee, Scotland, U.K.
†Institute for Genetics, University of Cologne, Cologne, Germany
‡College of Medicine, Dentistry & Nursing, University of Dundee, Dundee, Scotland, U.K.

Biochemical Journal www.biochemj.org

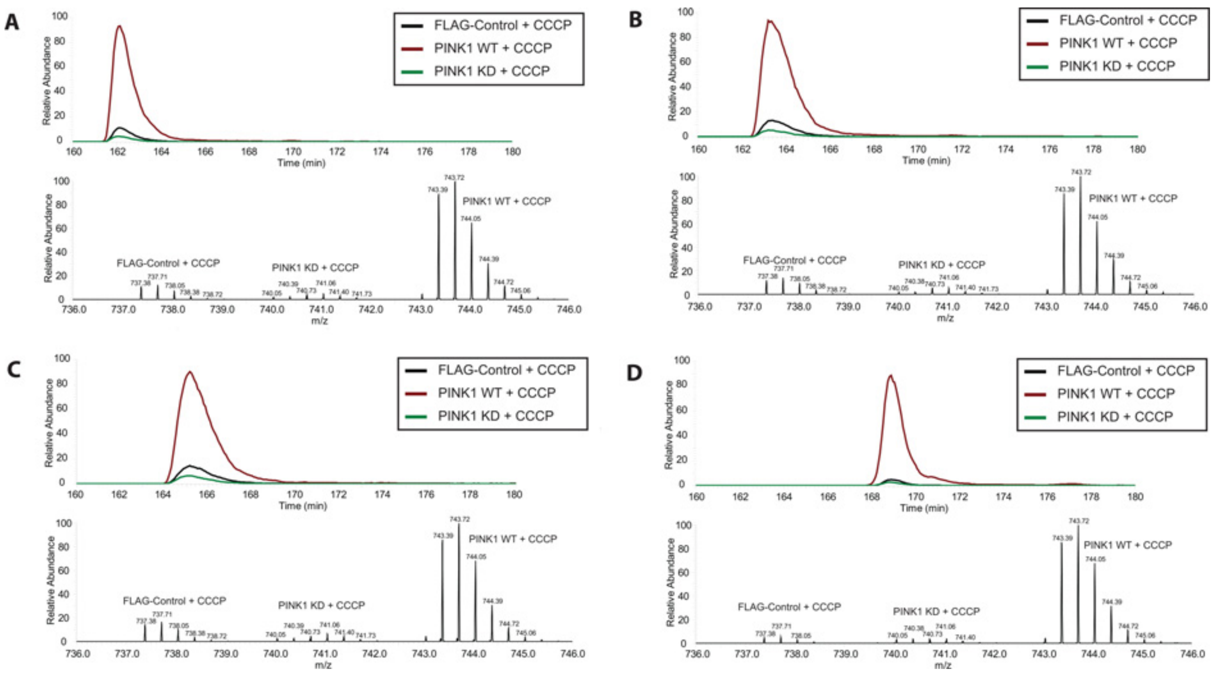

Figure S1 Reproducible phosphorylation of ubiquitin upon PINK1 stimulation by CCCP *in vivo*

Flp-In T-Rex HEK-293 cells stably expressing FLAG-empty, wild-type PINK1–FLAG or kinase-inactive PINK1–FLAG were grown in light, heavy and medium SILAC media respectively. Cells in each condition were stimulated with 10 μM CCCP for 3 h. Subsequently, membrane fractions were enriched by ultracentrifugation and solubilized in 1 % RapiGest. Lysates from each of the three conditions were mixed at 1:1:1 and digested with trypsin before phosphopeptide enrichment by HILIC and TiO_2, and analysis by MS. Data analysis was performed using MaxQuant. (**A**–**D**) Extracted ion chromatograms representing the ubiquitin Ser[65] phosphopeptide TLSDYNIQKEpSTLHLVLR in the three SILAC-labelled conditions over all four biological replicates. There is a ∼14-fold increase in abundance in cells transfected in the wild-type PINK1–FLAG compared with the kinase-inactive PINK1–FLAG.

[1] Correspondence may be addressed to either of these authors (a.kazlauskaite@dundee.ac.uk or m.muqit@dundee.ac.uk).

© 2014 The Author(s)
The author(s) has paid for this article to be freely available under the terms of the Creative Commons Attribution Licence (CC-BY) (http://creativecommons.org/licenses/by/3.0/) which permits unrestricted use, distribution and reproduction in any medium, provided the original work is properly cited.

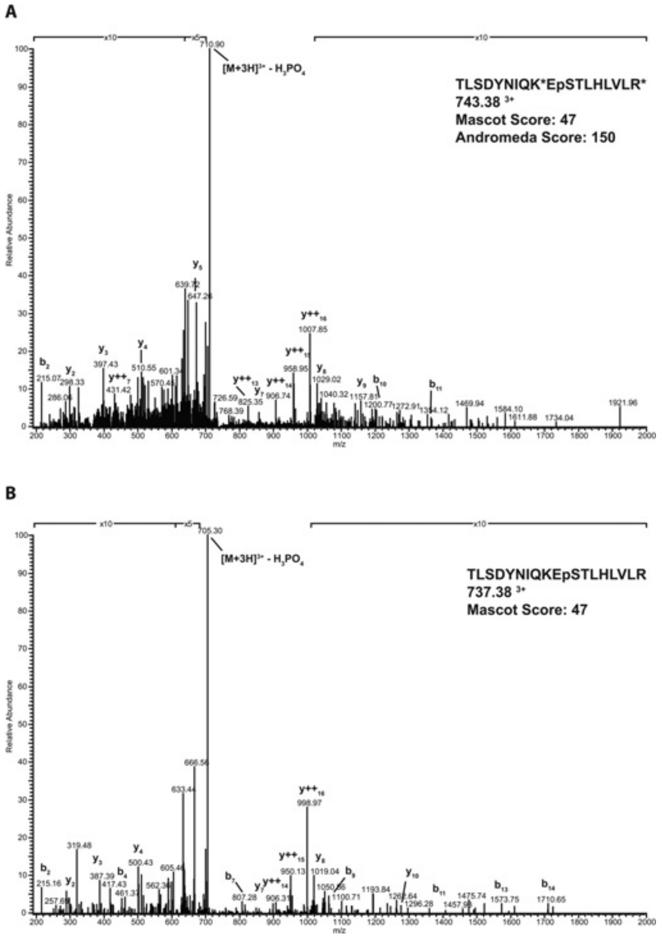

Figure S2 MS/MS fragmentation spectra of the phosphorylated ubiquitin peptide TLSDYNIQKEpSTLHLVLR

(**A**) MS/MS spectrum of the heavy (R10K8)-labelled peptide TLSDYNIQKEpSTLHLVLR identified in wild-type PINK1–FLAG-transfected cells. (**B**) Virtually identical MS/MS spectrum of TLSDYNIQKEpSTLHLVLR from a digest of ubiquitin *in vitro* phosphorylated by TcPINK1. Note that the doubly charged ion $[1105.5650]^{2+}$ of the same peptide was also positively identified in both experiments with Andromeda and Mascot Scores of 202 and 54 respectively.

Received 12 March 2014/24 March 2014; accepted 25 March 2014
Published as BJ Immediate Publication 25 March 2014, doi:10.1042/BJ20140334

© 2014 The Author(s)
The author(s) has paid for this article to be freely available under the terms of the Creative Commons Attribution Licence (CC-BY) (http://creativecommons.org/licenses/by/3.0/) which permits unrestricted use, distribution and reproduction in any medium, provided the original work is properly cited.
